# Efflux Pump Inhibitors in Controlling Antibiotic Resistance: Outlook under a Heavy Metal Contamination Context

**DOI:** 10.3390/molecules28072912

**Published:** 2023-03-24

**Authors:** Thi Huyen Thu Nguyen, Hai Dang Nguyen, Mai Huong Le, Thi Thu Hien Nguyen, Thi Dua Nguyen, Duc Long Nguyen, Quang Huy Nguyen, Thi Kieu Oanh Nguyen, Serge Michalet, Marie-Geneviève Dijoux-Franca, Hoang Nam Pham

**Affiliations:** 1 Department of Life Sciences, University of Science and Technology of Hanoi, Vietnam Academy of Science and Technology, 18 Hoang Quoc Viet, Cau Giay, Hanoi 10072, Vietnam; 2 Saint Paul Hospital, 12 Chu Van An, Hanoi 11114, Vietnam; 3 Department of Academic Affairs, University of Science and Technology of Hanoi, Vietnam Academy of Science and Technology, 18 Hoang Quoc Viet, Cau Giay, Hanoi 10072, Vietnam; 4 Institute of Natural Products Chemistry, Vietnam Academy of Science and Technology, 1H Building, 18 Hoang Quoc Viet, Cau Giay, Hanoi 10072, Vietnam; 5 Institute of Biological and Food Technology, Hanoi Open University, 101B Nguyen Hien, Hanoi 11615, Vietnam; 6 UMR 5557, Ecologie Microbienne, CNRS, INRAe, VetagroSup, UCBL, Université de Lyon, 43 Boulevard du 11 Novembre, F-69622 Villeurbanne, France

**Keywords:** multi-drug resistance, antibiotic resistance genes, efflux pump inhibitors, metal contamination, soil

## Abstract

Multi-drug resistance to antibiotics represents a growing challenge in treating infectious diseases. Outside the hospital, bacteria with the multi-drug resistance (MDR) phenotype have an increased prevalence in anthropized environments, thus implying that chemical stresses, such as metals, hydrocarbons, organic compounds, etc., are the source of such resistance. There is a developing hypothesis regarding the role of metal contamination in terrestrial and aquatic environments as a selective agent in the proliferation of antibiotic resistance caused by the co-selection of antibiotic and metal resistance genes carried by transmissible plasmids and/or associated with transposons. Efflux pumps are also known to be involved in either antibiotic or metal resistance. In order to deal with these situations, microorganisms use an effective strategy that includes a range of expressions based on biochemical and genetic mechanisms. The data from numerous studies suggest that heavy metal contamination could affect the dissemination of antibiotic-resistant genes. Environmental pollution caused by anthropogenic activities could lead to mutagenesis based on the synergy between antibiotic efficacy and the acquired resistance mechanism under stressors. Moreover, the acquired resistance includes plasmid-encoded specific efflux pumps. Soil microbiomes have been reported as reservoirs of resistance genes that are available for exchange with pathogenic bacteria. Importantly, metal-contaminated soil is a selective agent that proliferates antibiotic resistance through efflux pumps. Thus, the use of multi-drug efflux pump inhibitors (EPIs) originating from natural plants or synthetic compounds is a promising approach for restoring the efficacy of existing antibiotics, even though they face a lot of challenges.

## 1. Introduction

The widespread use of antibiotics in therapeutics and agriculture has led to an increase in antibiotic resistance genes (ARGs) [1]. Moreover, pollution caused by heavy metals (HMs) has become a serious concern, especially in developing countries. HMs, such as Cu, Zn, Cd, Ni, Fe., etc., are natural components of the Earth’s crust, but their concentrations have reached levels that are toxic for living organisms as a result of anthropogenic activities, such as mining and the metallurgical industry or agricultural practices, thus affecting the overall functioning of ecosystems. In order to deal with these situations, microorganisms use an effective strategy that includes a range of expressions based on biochemical and genetic mechanisms [2]. Recently, more evidence suggests that heavy metal contamination can also affect the dissemination of ARGs [1].

**Figure 1:**
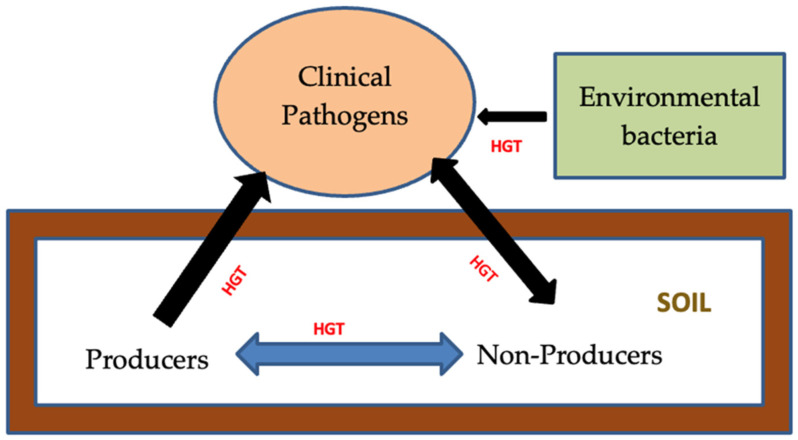
Pathways in which antibiotic resistance genes transfer from the natural environment to the clinical environment (inspired from [8]).

There are two significant mechanisms of antibiotic resistance: intrinsic and acquired resistance. The intrinsic mechanism, also known as natural resistance, is caused by a structural gene. On the other hand, acquired resistance is obtained by transformation, transposition, and conjunction and is called horizontal gene transfer (HGT) [3]. Horizontal gene transfer is a deciding factor that mediated the antibiotic resistance between bacteria in the environment and in hospitals [4]. Pollution can increase the production of reactive oxygen species, leading to mutations in the chromosomal DNA of bacteria under stress [5,6]. Similarly, antibiotics are widely used in the agriculture and pharmaceutical industries or in hospital waste, all of which contaminate the environment [7]. In the context of antibiotic resistance, the acquired mechanisms could become more severe because resistance determinants may be mediated by plasmids in the acquisition of genetic material [5,8]. At present, it is widely recognized that environmental factors contribute to the acquired resistance of pathogenic bacteria through four phases: the emergence of novel resistance genes, mobilization, transfer to pathogens, and dissemination [8]. On the other hand, the acquired resistance mechanisms are generally obtained because of HGT and include plasmid-encoded specific efflux pumps (such as *TetK* and *TetL* of *S. aureus*). Recently, the literature has suggested that the resistance genes of bacteria in the environment could play an important role in the evolution of antibiotic resistance in pathogens [8]. Through HGT, the bacteria in pathogens may acquire resistance genes from the reservoir of ARGs that are encountered in clinical environments, as well as natural environments (Figure 1) [9]. Furthermore, the evidence for co-selection between metal and antibiotic resistance in the environment originated from diverse habitats contaminated with various metals [10]. The selection of resistance in successive bacterial generations could be the result of using antimicrobials at trace levels. This selection engenders hypermutable strains that increase the highly acquired resistance and foster the transposition of mobile genetic elements [5].

## 2. Co-Selection of Antibiotic and Metal Resistance Mechanism

**Figure 2:**
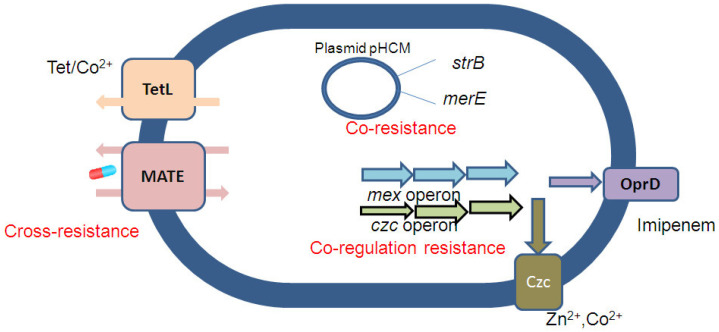
Examples of molecular mechanisms that underline metal and antibiotic co-selection (inspired from [10]).

The data from numerous studies has shown that antibiotic-resistant bacteria increase in highly anthropogenic environments. Various chemical stressors, such as antibiotics, biocides, hydrocarbons, and metals, may contribute to this selection. Significantly, the presence of multi-drug resistant bacteria in animals is also frequently reported and must be monitored in farm animals regarding the risk of spread from animals to humans [11,12,13]. There is increasing evidence that metal-contaminated soil acts as a selective pressure, favoring antibiotic resistance by the co-selection of resistance mechanisms [14]. The exposure to metals could co-select for both metal and antibiotic resistance in the same bacterial cell. This resistance can be co-selected by different processes: (i) co-resistance, where different resistance genes are physically located on the same genetic element; (ii) cross-resistance, where the presence of the same genetic determinant confers resistance to both antibiotics and metals; and (iii) co-regulation, where the metal or antibiotic acts as an inducer of a common regulatory system that is responsible for the expression of diverse metal and antibiotic resistance determinants (Figure 2) [10,15]. In the mechanism of co-selection between heavy metal and antibiotic resistance, several studies have reported the correlation of heavy metal concentrations with ARGs in water, soil, or sediment when bacteria were exposed to both heavy metals and antibiotics [14]. Based on in situ monitoring in the soil of northern China, data has demonstrated the correlation between *tetA* and *tetB*/*P* (tetracycline resistance genes) with Zn concentration, *sulII* (sulfonamide resistance genes), and *copB* (Cu resistance genes); or the connection between Cd and Zn with macrolide and aminoside in diverse environments [1,16]. Moreover, co-selection has been shown in bacterial communities in humans, thus indicating significant implications for hospitals and environments that are contaminated with heavy metals [17,18]. The occurrence of heavy metal resistance genes, including *pcoA*, *merA*, *silC*, and *arsA*, was found in clinical multi-drug resistant Enterobacteriaceae with *bla*_NDM-1_ (genes responsible for all antibiotic resistance except colistin and fluroquinolones) and *bla*_CTX-M-15_ (genes encoded extended-spectrum β-lactamases) [19,20].

It has been reported that Cu in a contaminated environment co-selected with strains resistant to Cu and various antibiotics. These strains exhibit significantly stronger resistance to tetracycline, acid nalidixic, ampicillin, and chloramphenicol than susceptible strains. Moreover, certain concentrations of heavy metals, such as Hg, Zn, or Cd, in soil or water may trigger co-selection mechanisms (i.e., the “minimum co-selective concentration”) [14]. Additionally, several studies found that the impact of Pb and Zn contamination resulted in the abundance of opportunistic pathogens in the root endosphere of the *Pteris vitata* plant. Among them, the *Burkholderia cepacia* complex and *Ralstonia pickettii* strains were isolated, which have structured multi-efflux pumps [21]. In addition, *Cupriavidus* is a highly resistant genus and was the only one found in elevated proportions at two mining sites. Since it does not present in non-contaminated *P. vittata* rhizospheric soils, *Cupriavidus* could then be involved in the adaptation process of the plant under metal stress [22]. Another study on the bacterial population of the *Miscanthus x gigantus* plant from agricultural soil in France found the selection of *Stenotrophomonas* and *Pseudomonas* under metal pressure [23].

Co-resistance occurred in the methicillin-resistant *Saphylocccocus aureus* (MRSA), which accumulated resistance mechanisms to heavy metals and other antibiotics, such as fluoroquinolone and carbapenem [24,25]. Co-resistance plays an essential role in co-selection because the genetic liaison for co-resistance includes different mobile genetic elements, such as transposons, integrons, or plasmids [25].

When different antimicrobial agents, such as metals and antibiotics, attack the same target, a second potential co-selection mechanism, called cross-resistance, may be observed [3,26]. Cross-resistance could manifest in efflux systems that are resistant to both heavy metals and antibiotics [10,27] (Table 1). In *Listeria monocytogenes*, the *MdrL* gene encodes multi-drug efflux proteins that pump Zn, Co, and Cd, as well as erythromycin and clindamycin [28].

Co-regulation, the third mechanism involved in maintaining and proliferating antibiotic resistance, causes a range of transcriptional and translational responses to metal or antibiotic exposure in order to form a coordinated response [10]. Perron et al. highlighted the Zn–antibiotic resistance co-regulation in Gram-negative *Pseudomonas aeruginosa*, in which the elevated expression of resistant Zn and the decreased porin pathway lead to increased imipenem resistance [29].

Cross-resistance and co-regulation mechanisms are specifically related to efflux pumps [30]. Moreover, metal resistance is also similar to the mechanism of antibiotic resistance in bacteria, as seen in Table 1 [7].

Soil microbiota is considered a reservoir of resistance genes available for exchange with pathogenic bacteria. Forsberg et al. demonstrated that multi-drug resistant bacteria in the soil could confer resistance to five classes of antibiotics; they were able to perfectly identify the nucleotides in genes from different human pathogens [31]. Antibiotic resistance caused by efflux mechanisms were found in cultivable bacteria in soil, such as *Pseudomonas*, *Stenotrophomonas*, *Sphingobacterium*, and *Chryseobacterium* [32]. In some soil and water samples polluted with heavy metals, a certain prevalence of antibiotic resistance strains was identified, thus suggesting that the exposure of bacteria to increasing metal contamination can lead to the co-selection of antibiotic resistance [7]. However, whether antibiotic efflux-pump-carrying bacteria are favored in heavy-metal-contaminated environments has not yet been elucidated [33].

## 3. Antibiotic Resistance Caused by Efflux Pump Mechanisms

Antibiotics have opened a new era in medicine and the pharmacological industry with the discovery of penicillin and streptomycin in the 20th century; from this, deadly bacterial infections were easily treated [34]. In order to deal with antibiotics, bacteria adapt and overcome treatments; this is called antibiotic resistance. In 2014, the WHO described how the levels of bacteria with resistance to available antibiotics was impacting infection treatments in hospitals and communities. In fact, there is strong evidence showing that the antibiotics that are widely used for infection treatment in animals and humans are mainly responsible for the emergence of bacteria with resistance mechanisms [35].

**Figure 3:**
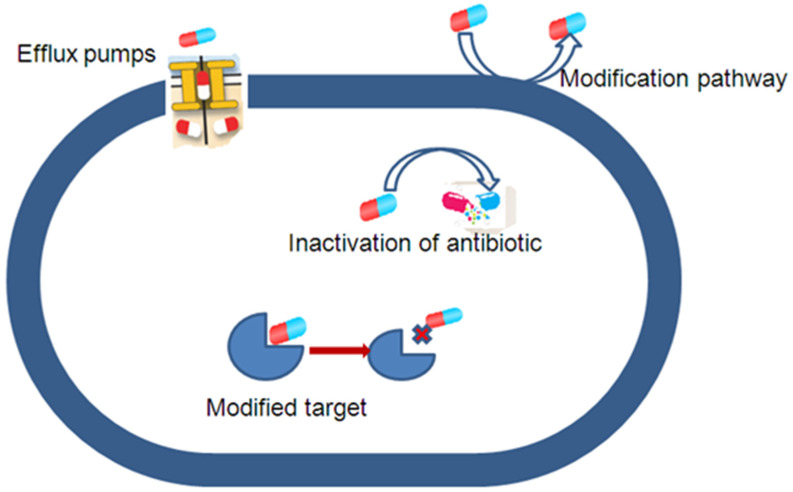
Major mechanisms of bacteria resistant to antibiotics (inspired from [34]).

Multi-resistance to antibiotics, defined as resistance to at least three different classes of antibiotics, is a growing concern in hospitals and represents a serious threat to patients [36]. It is worth noting that four general antibiotic resistance mechanisms were reported: the inactivation or modification of antibiotics, modification of antibiotic targets, modification of metabolic pathways, and reduction in the intracellular accumulation of antibiotics by decreasing membrane permeability and/or increasing the activity of efflux systems (Figure 3). Efflux pumps are transport proteins in bacteria that are responsible for the extrusion of substrates, such as antibiotics, metals, etc., from the internal to the external cellular environment [34]. These efflux systems are important in prokaryotes and catalyze the active efflux of various compounds, including antibiotics, organochlorine compounds, and metals [30]. Some efflux pumps may selectively extrude specific antibiotics, but others may associate with various substrates called MDR pumps [37,38].

In 1980, it was first discovered that bacteria could efflux antibiotics in a study on the tetracycline resistance mechanism [38]. After that, developments in molecular microbiology have discovered the features of many efflux pumps in both Gram-negative and Gram-positive bacteria that largely contribute to the acquisition of the MDR phenotype [38,39,40]. MDR efflux pumps have six main physiological features that involve antibiotic resistance: (1) they are in every living cell (omnipresence); (2) each species has the same efflux pumps (are efflux pumps the same or are they different isoforms/variants?); (3) various efflux pumps are found on a single cell; (4) one pump can have high binding affinity to multiple substrates; (5) the expression of efflux pumps is highly regulated; and (6) the compounds produced by hosts, such as plant signals or bile salts, could cause the expression of efflux pumps [41].

**Figure 4:**
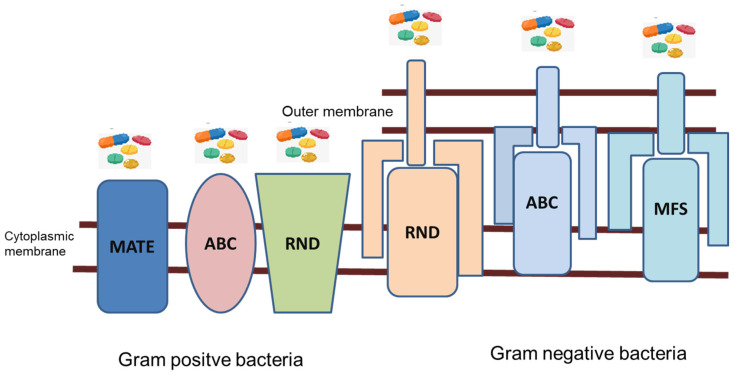
Major efflux pump families (inspired from [34]).

These efflux pumps could be found in 6 to 18% of membrane transporter proteins. These efflux pumps are energy dependent because the transport substrates must fight the concentration gradient. Thus, these efflux pumps could be classified into two groups (primary and secondary transporters) based on the mechanism by which they derive energy. The primary transporter used energy from ATP binding and hydrolysis for efflux, which is named ABC (ATP Binding Cassette). The secondary groups draw energy from electrochemical gradients, such as Multidrug and Toxic Compound Extrusion (MATE), Major Facilitator Superfamily (MFS), Staphylococcal Multi Resistance (SMR), and Resistance Nodulation Division (RND) (Figure 4) [30,34,37,42]. MFS is the largest group of efflux pump systems, with substrates including glucoses, neurotransmitters, amino acids, cyclical Krebs metabolites, and drugs. Most MFS pumps contain 400–600 amino acids and have 12–14 transmembrane protein chains. MFS is also the most relevant in Gram-positive bacteria [43]. Common MFS pumps in bacteria include tetracyclin-boosting TetA pumps, EmrE (*E. coli*), NorA, and QacA (*Staphylococcus aureus*) [43,44].

The RND family is the most clinically important of the Gram-negative bacteria; they have been reported as a result of regulatory mutations in MDR Gram-negative bacteria because of the increased expression of chromosomal encoding in them [37].

Reportedly, more than one pump can perform antibiotic efflux, such as *Pseudomonas aeruginosa*, which is known as an opportunistic pathogen in various infections and expresses more than ten efflux pumps in the RND family, in which at least five exhibit various levels of antibiotic resistance in clinical settings (Table 2) [37,45]. Efflux pumps are also resistant to heavy metals. Some studies report that a specific efflux pump involves heavy metal and antibiotic resistance. For instance, the overexpression of CzcCBA pumps (resistance to Cz, Cb, and Co) by *Pseudomonas aeruginosa* correlated with the level of heavy metals. However, a system two component regulator expressed not only CzcCBA but also OprD porin, which transported carbapenem inside the bacteria. However, OprD is related to Mex-T. Efflux pumps are a well-established resistance mechanism that maintain the internal metal ion concentration and homeostasis in the cell. Various strains resist Cu, Co, As, Zn, Cd, tetracycline, chloramphenicol, and β- lactams through the rapid efflux of metals or antibiotics.

MDR pumps are not only limited in the clinical environment but also in natural ecosystems, such as soil or the rhizosphere, and the non-clinical cross-interaction between plants and bacteria. Besides, environmental factors, such as oxidative stress or antibiotics, can cause efflux pump overexpression. For example, *SmDEF* pumps, which are responsible for resistance to quinolone in *Stenotrophomonas malltophilia*, experience elevated protein expression caused by the binding of flavonoid to its transcriptional repressor *SmeT* [49,50]. Moreover, *S. malltophilia* is a Gram-negative bacteria sourced from the ecosystem that performs the role of an opportunistic pathogen. They appear in both natural and anthropogenic environments, such as soil contaminated with heavy metal, but they cause numerous infections in hospitals. Several studies have shown that isolates of *S. maltophia* from clinical and natural environments are phenotypically multi-drug resistant and are taxonomically homogeneous [13,42]. *S. maltophilia* expresses various resistance mechanisms, which has led to their intrinsically low susceptibility to antibiotics. Among their mechanisms, multi-drug efflux pumps are major contributors to MDR phenotypes [41]. In addition, the function of efflux pumps is not only limited to antibiotic resistance, but also plays a role in plant–bacteria interactions, or detoxify both intermediate metabolites and toxic factors, such as heavy metals or antibiotics [42,48]. Nevertheless, despite the potential role of these genes in natural, non-clinical ecosystems, it is a fact that they contribute to the success of *S. maltophilia* when it causes an infection in a treated patient. Alongside their role in protecting strains against antimicrobials, efflux pumps are physiological functions of bacteria. To clarify these roles, it is essential to understand these efflux pumps in the non-clinical context of opportunistic pathogens that have the tendency to quickly develop new antibiotic resistance. In particular, knowledge of these roles helps to create more powerful drugs for clinical practice with the potential for commercial exploitation, and even to develop more sustainable biological processes [41,47].

## 4. Secondary Metabolites Production of Plants as Potential EPIs

Plants possess two main adaptive strategies used to cope with an excess of heavy metals: (i) exclusion, which is the most commonly used strategy, where the metal concentrations in shoots are kept constantly low while the external concentrations vary, or (ii) accumulation, where metal is more translocated in shoots than in roots, with a distinction between indicators that concentrate metals at levels close to that of soil and hyperaccumulators that are able to accumulate metals at very high levels [22,23,51].

The modification of this metabolic production of plants could have a direct impact (production of metabolites with antimicrobial activities) or indirect impact (production of metabolites affecting the bioavailability of metals) on the function and diversity of bacterial populations in soils, particularly those associated with roots (rhizosphere) or leaves (phyllosphere). Due to the complexity of the interactions involved and multiple matrices (soil/roots), the impact of secondary metabolites synthesized by plants on soil bacterial populations remains elusive. However, plants can structure bacterial communities of soils mainly through their root exudates [22,23,52]. Secondary metabolites are chemical compounds manufactured by plants to help them compete in their environment [53]. The biological effects of secondary metabolites provide scientific justification for using plant medicine for their antibiotic, antifungal, and antiviral effects. The main groups of secondary plant metabolites, many of which function as EPIs, include phenolics, alkaloids, saponins, terpenes, lipids, and carbohydrates that have been identified using analytical equipment, such as HPLC and MS/MS (Table 2) [54,55]. Exposure to Cu significantly increased the synthesis of phenolic compounds in the shoots of *Imperata cylindrica*, which is a grass in Chile that grows in soil with varying distributions of Cu contamination [56]. Under stress conditions, phenols act as chelating metals through hydroxyl and carboxyl groups and inhibit lipid peroxidation by trapping alkoxyl radicals. Therefore, exposure to toxic elements leads to the formation of reactive oxygen species (ROS), which consequently increases the production of phenolic compounds in plants [56]. Phenolics from root exudation depend on metal concentrations or plant species [57].

A theory by Thjis called “Plant call for support” hypothesized that pollution-induced changes in root exudation could lead to the selection of microbial communities with beneficial traits for plants (e.g., degradation capabilities), thus allowing for better adaptation to the presence of these contaminants [58]. On the other hand, plant-derived antimicrobial compounds are sometimes MDR pump substrates [59,60]. Plants also produce metabolites that can induce the expression of bacterial efflux pumps, allowing bacteria to effectively colonize the rhizosphere and influence MDR phenotypes by inhibiting the activity of efflux pumps in order to reduce the MIC of their antimicrobial substrates [60,61]. Since plants and rhizosphere regions share close relationships, plants may have developed specific strategies to inhibit or promote these microorganisms, thus producing a source of potentially bioactive compounds, including antimicrobial agents or the inhibitors of efflux pumps [59,61]. There are different strategies that inhibit efflux pumps: (1) develop new antibiotics that are not affected by efflux pumps; (2) change the function of efflux pumps through structural modifications; (3) inhibit the energy sources needed to operate efflux pumps; (4) decrease the expression of efflux pump genes; and (5) competitively/non-competitively inhibit efflux pumps [62]. By testing a resistant strain that is susceptible to the antibiotic, in vitro experiments have elucidated that EPIs can restore the efficacy of an antibiotic and reduce the Minimum Inhibitory Concentrations (MICs) of antibiotics and the opportunities for resistant growth when associated with the antibiotics [61,62,63].

*Ocimum tenuiflorum* or *Phyllanthus amarus* were cultivated in Cr-polluted soil, resulting in a significant increase in phenolic compounds, such as eugenol or hypophyllathin [64]. Moreover, eugenol was reported as having an inhibitory effect on NorA efflux pumps when combined with norfloxacin in *S.aureus*. Eugenol was also demonstrated to inhibit the *arB* gene with cefotaxim and ciprofloxacin in ESBL *Enterobacteriaceae* [65,66].

*Fallopia japonica*, also called Japanese knotweed, is known as a plant that can grow in metal-polluted soil. UV, MSMS, and HRMS spectra analyses identified a dihydroanthracenone derivate from *F. japonica* extract known as torosachrysone. Other anthracenes derivatives, including anthraquinone and dianthrone, were also identified. The proportion of these compounds from *F. japonica* root extract were found to be increased with metal contamination [51]. Emodin is a bioactive compound identified from the root extract of *F. japonica*. A recent study in 2020 reported a correlation between the abundance of rhizospheric *Stenotrophomonas* and the concentration of emodin. This compound protects plants against toxic factors, but also possess antimicrobial effects. The research showed that emodin could assist *F. japonica* to grow under a root endosphere enriched with *Stenotrophomonas* [67]. *F. japonica* roots have bioactive compounds, including anthraquinon, flavonoid, and stilbene, which have related pharmacological effects. Resveratrol, physcion, and stilbene were also identified in *F. japonica* roots. They were reported as playing an important role in antibacterial activity tests against *Bacillus cereus*, *Listeria monocytogenes*, *S. aureus*, and *Salmonella anatum*. Another in vitro study also demonstrated the potent inhibitory effect of emodin from an extract of *F. japonica* toward Gram-negative *Haemophilus parasuis* [68]. The interactions between emodin and efflux transporters indicated that emodin might be the substrate of P-glycoprotein and the multi-drug-resistant-associated protein MRP2. Most recently, Nguyen et al. revealed that the major component of *F. japonica’s* subterraneous part, including emodin, emodin dianthrone, physcion, torosachrysone, and fallopion isomers, exhibited potential efflux pump inhibitory activity against *S. maltophilia* BurA1 and BurE1 strains [61]. These results will be helpful to explain the drug–drug interaction mechanisms between emodin and other drugs and provide the basic data for clinical combination therapy. Indeed, emodin, as well as baicalin, schizandrin, and berberine, can be used to decrease *hefA* mRNA expression in order to reduce the MICs of amoxicillin and tetracycline against some multi-drug resistant *Helicobacter pylori* [69]. In addition, resveratrol extracted from the *Naculea pobeguinii* plant showed a synergic effect with streptomycin in *Klebsiella pneumonie* [70].

*Pteris vittata*, which is known as a metal hyperaccumulator, lives in tropical and subtropical areas, such as China, Southeast Asia, Africa, or Australia [22]. Moreover, *P. vittata* can grow and develop normally on Zn-contaminated soil because it can accumulate Zn in leaves up to 737 mg/kg. In Vietnam, *P. vittata* and some other ferns have been used to remedy metal-contaminated soil in the Thai Nguyen mine with plants [22,71,72]. In 1999, Singh et al. reported that a decoction of *Pteris vittata* was effective in the treatment of dysentery [63]. In 2008, Singh et al. also showed that this fern contains rutin, which is known to have the ability to fight microorganisms that cause gastrointestinal diseases [64]. In addition, some other studies have also described the composition of *P. vittata*, with glycosides of apigenin, luteolin, quercetin, kaempferol, leucocyanidin, and leucodelphinidin [65]. In the pharmacological field, several studies have shown that P. vittata has antioxidant, hypoglycemic, anti-inflammatory, antiplatelet, and anticancer activities [72,73,74]. Recently, Nguyen and coworkers revealed the mechanism of metal accumulation in *P. vittata* dominating the Thai Nguyen mining sites. Secondary metabolites of *P. vittata* were identified, including 70 flavonoids, 14 benzoic acids, 16 phenylpropanoic acid derivatives, 2 stilbenoids, 9 phenols, 20 alkaloids, 5 coumarins, and 3 terpenoids [75]. Among them, quercetin has shown remarkable properties as an antibacterial agent and an EPI. When co-administered with other compounds, it increased the oral bioavailability of digoxin in pigs, moxidectin in sheep, and paclitaxel in rats [76,77,78]. The antimicrobial activity of luteolin, kaempferol, and apigenin were also reported [69]. Additionally, several studies found that the impact of Pb and Zn contamination resulted in an increase in the abundance of opportunistic pathogens in the rhizosphere of *P. vittata*. Among these bacteria, the *Burkholderia cepacia* complex and *Ralstonia pickettii* strains were isolated, both of which have diverse structured efflux pumps [21].

A large number of phytochemicals, including flavonoid or terpene, are substrates of efflux pumps in the defense against bacterial infection and are considered as potential sources for novel EPIs (Table 3) [44,54,61]. A recent systematic review by Dias and others searched 75 different terpene compounds that have been reported as EPIs against both Gram-positive and Gram-negative bacteria. Among them, *S. aureus* with *NorA* was the most investigated [79]. Additionally, a large number of alkaloids from various origins were found among the phyto-EPIs [80]. Flavonoid was also identified as inhibiting efflux pumps; this included chalcones, fla-van-3-ols, flavanones, flavones, flavonols, flavonolignans, and isoflavones [81]. Epigallocatechin gallate, which is known as polyphenol, was affected in *Campylobacter* spp. when combined with tetracycline, erythromycin, and ciprofloxacin. However, they were observed at high concentrations as EPIs. Accordingly, the major question is whether metal contamination could enhance the efflux pump inhibitor concentration extracted from plants that were grown in metal-polluted soil.

## 5. Challenges and Future

Biocides used in agricultural settings and widely used antiseptics both contain toxic metals. Thus, increasing antibiotic resistance is of particular concern in both agricultural and clinical environments. However, the majority of observational studies performed to demonstrate co-selection at the population and community level were primarily based on analyzing the culture-dependent phenotypes of isolates. Therefore, it is essential to evaluate the role of metal contaminants as a selective force for antibiotic resistance determinants in the environment at a biological level [10]. The studies identified efflux determinants of resistance that serve to identify potential resistance mechanisms using cloned genes and in vitro strain construction, but the clinical significance of many known efflux determinants is yet to be demonstrated [39,82].

Regarding the importance of efflux-related MDR, it is essential to develop a new therapeutic approach target to inhibit bacterial efflux pumps in order to fight bacteria with over-expressed MDR efflux systems. Thus, EPIs tend to solve antibiotic resistance in the treatment of infections. However, its chemical existence will have to pass rigorous criteria in order to become a successful EPI. There are five important factors that determine the availability of an EPI: they must not be antibacterial, as this can increase the fighting of these EPIs through resistance mechanisms; EPIs should be selective and should not have pharmacological activity on eukaryotic efflux pumps; they should have a good therapeutic index and pharmacokinetic profile; they need to be highly safe, as well as non-toxic; they should be stable in serum in order to ensure high concentrations for treating infections; and, finally, in order to be successful at a commercial level, the production of the EPI must be economically feasible [62].

The importance and advantages of EPIs offer many potential benefits as therapeutic agents, but they face a lot of challenges. This strategy will save a lot more time, effort, and capital than the discovery of new antibiotics. However, studying a new chemical entity similar to EPIs will require lots of time and money [34,83].

Numerous studies have been conducted to investigate EPIs from plant and synthetic compound origins, but they have only been realized at the laboratory level. The structure of naturally derived EPIs is not only complicated and bulky but also extremely difficult to synthesize. On the contrary, synthetic products are simpler to synthesize but they have poorer solubility and toxicity and are disadvantageous for cell permeability. As a consequence, if EPIs are an available drug for treatments with different indications by other mechanisms, they would have potential for academic, scientific, and pharmaceutical industrial strategies [34,38]. Recently, several studies have been performed at different stages to identify the effect of EPI phytomolecules in enhancing the antibiotic susceptibility of pathogenic bacteria. This pathway may lead to clinical phases and clinical practices, so it is necessary to perform more comprehensive studies to search for EPI phytomolecules [84].

In summary, there has been a considerable amount of progress in the research, but a lot more work is necessary before finally approving the clinical use of EPIs.

## 6. Conclusions

In metal-contaminated soil, co-selection between heavy metal and antibiotic resistance has increased more rapidly and was more concerned with the reservoirs of available ARGs for exchange among pathogenic agents. Co-selection resistance has three main mechanisms, but two of them specifically involve efflux pumps. However, whether antibiotic efflux-pump-carrying bacteria are favored in HM-contaminated environments has not yet been elucidated. In the context of heavy metal contamination, the secondary metabolites produced by plants to cope with it may be a potential source of efflux pump inhibitors. However, there are various challenges that must be overcome for EPIs to become a viable therapy for treatment.

## Figures and Tables

**Table 1:** Example of cross-resistance to both antibiotics and metals [10,27].

Bacteria	Metal Ions	Antibiotics	Mechanism of Cross-Resistance
*Burkholderia cepacia*	Cd, Zn	Ofloxacin, erythromycin, kanamycin, novobiocin, β-lactams	Same efflux pumps systems
*Salmonellaa Typhimurium*	Cu, Zn,	β-lactams	Same efflux pumps systems
*Listeria monocytogenes*	Co, Zn, Cd	Erythromycin, clindamycin	Same efflux pumps systems

**Table 2:** Examples of efflux pump systems in bacteria.

Bacteria	MFS	SMR	MATE	ABC	RND	Ref
*Straphylococcus aureus*	*Nor A*, *Nor B*, *Nor C*, *MdeA*, *LmrS*	*QacA*, *QacB*, *QacD*, *Ebr*	*MepA*	*MrsA*	*FarE*	[45]
*Klebsiella pneumoniae*	*kmrA*	*kpnEF*	*kdeA*		*acrAB*, *kexD*	[46]
*E. coli*	*EmrAB-TolC*, *Dep*	*EmrB* *Bcr* *EmrD* *EmrE*		*MacAB-TolC*	*AcrAD* *-* *TolC*	[47]
*Pseudomonas aeuruginosa*		*EmrE*	*PmpM*		*MexAB-OprM MexCD-OprJ MexEF-OprN MeXY-OprM* *MeCD-OprJ* *MexAB-OprM*	[45,48]

**Table 3:** EPIs originating from plants, their target, and the bacterial pathogens inhibited [44,54].

Plant	Active Compound	Bacterial Target	Efflux Pump Target
*Caesalpinia spinosa*	Tannic acids	*Acinobacteria baumanii*	*Yo*
*Eucalyptus tereticornis*	Ursolic acid and derivatives	*E. coli*	*AcrAB*, *TolC*, *MacB*, and *YojI*
*Terminola chebula*	Gallotannin 1,2,6-tri-O- galloyl-β-D	*E. coli*	*ND*
*Thymus vulgaris*	Baicalein	*Salmonella enteridis*	NorA
*Dalea versicolor*	Flavanoid, Phenolic	*Staphylococcus aureus*, *Bacillus cereus*	*NorA*
*Berberis* spp.	Berberin và Palmatin	*Staphylococcus aureus*, *Pseudomonas aeruginosa*	*NorA*, *MexAB-OprM*
*Fallopia japonica*	Resveratrol	*Mycobacterium* spp.	*TetK*

## Data Availability

Not applicable.
